# Supplementary material for: Localization of KRAS downstream target ARL4C to invasive pseudopods accelerates pancreatic cancer cell invasion
Source: eLife. 2021 Sep 30;10:e66721. doi: 10.7554/eLife.66721 (PMC8598236; doi:10.7554/eLife.66721)
Supplement: Supplementary file 1. [file elife-66721-supp1.docx]

**Supplementary Information**

**Localization of KRAS downstream target ARL4C to**

**invasive pseudopods accelerates**

**pancreatic cancer cell invasion**

**Akikazu Harada^1,4^, Shinji Matsumoto^1,4^, Yoshiaki Yasumizu^3,4^,**

**Toshiyuki Akama^1^, Hidetoshi Eguchi^2^, and Akira Kikuchi^1*^**

Departments of ^1^Molecular Biology and Biochemistry, ^2^Gastroenterological Surgery, Graduate School of Medicine, ^3^Laboratory of Experimental Immunology, WPI Frontier Immunology Research Center, ^4^Integrated Frontier Research for Medical Science Division, Institute for Open and Transdisciplinary Research Initiatives (OTRI), Osaka University, 2-2 Yamadaoka, Suita, Osaka 565-0871, Japan

*****Correspondence author. Department of Molecular Biology and Biochemistry, Graduate School of Medicine, Osaka University, 2-2 Yamadaoka, Suita 565-0871, Japan. Phone, 81-6-6879-3410. Fax, 81-6-6879-3419. E-mail: akikuchi@molbiobc.med.osaka-u.ac.jp

Supplementary file 1 Tables 1-8.

**Supplementary File 1 Table 1.** Clinicopathological features of high and low ARL4C expression in PDAC.

| **Parameters** |  | **High ARL4C cases** | **Low ARL4C cases** | ***P* value** |
| --- | --- | --- | --- | --- |
| Stage | IA-IIA | 20(76.9%) | 6 | 0.49 |
|  | IIB-III | 27(87.1%) | 4 |  |
| T classification | T1/2 | 6(66.7%) | 3 | 0.18 |
|  | T3 | 41(85.4%) | 7 |  |
| N classification | N0 | 20(76.9%) | 6 | 0.49 |
|  | N1 | 27(87.1%) | 4 |  |
| Lymphatic vessel invasion | ly0 | 13(72.2%) | 5 | 0.26 |
|  | ly1/2 | 34(87.2%) | 5 |  |
| Venous invasion | v0 | 28(82.4%) | 6 | 1 |
|  | v1/2/3 | 19(82.6%) | 4 |  |
| Perineural invasion | ne0 | 2(40.0%) | 3 | 0.033 |
|  | ne1/2/3 | 45(86.5%) | 7 |  |
| Age | <65 | 12(75.0%) | 4 | 0.44 |
|  | ≧65 | 35(85.4%) | 6 |  |
| Sex | Male | 24(80.0%) | 6 | 0.73 |
|  | Female | 23(85.2%) | 4 |  |
| Tumor location | Head | 32(84.2%) | 6 | 0.72 |
|  | Body and/or Tail | 15(78.9%) | 4 |  |

Associations between ARL4C expression and the clinicopathologic characteristics were investigated. *P* values were calculated using the Chi-square test. T1, tumor limited to the pancreas, 2 cm or less in greatest dimension. T2, tumor limited to the pancreas, more than 2 cm in greatest dimension. T3, tumor extends beyond the pancreas but without involvement of the celiac axis or the superior mesenteric artery. N0, no regional lymph node metastasis. N1, regional lymph node metastasis. ly0, no lymphatic vessel invasion. ly1, mild lymphatic vessel invasion. ly2, moderate lymphatic vessel invasion. v0, no venous invasion. v1, mild venous invasion. v2, moderate venous invasion. v3, severe venous invasion. ne0, no perineural invasion. ne1, mild perineural invasion. ne2, moderate perineural invasion. ne3, severe perineural invasion. See Supplementary File 1 Table 1-source data1.

**Supplementary File 1 Table 2.** The list of ARL4C binding proteins

| Band number | Identified Protein |
| --- | --- |
| 1 | PRKDC |
| 2 | IQGAP1 |
| 3 | IARS |
| 4 | DHX9, SMC3 |
| 5 | PARP1, MATR3, HNRNPU |
| 6 | NCL |

The list of ARL4C binding proteins was shown. See Supplementary File 1 Table 2-source data1.

**Supplementary File 1 Table 3.** Clinicopathological features of high and low IQGAP1 expression in PDAC.

| **Parameters** |  | **High IQGAP1 cases** | **Low IQGAP1 cases** | ***P* value** |
| --- | --- | --- | --- | --- |
| Stage | IA-IIA | 14(53.8%) | 12 | 1 |
|  | IIB-III | 17(54.8%) | 14 |  |
| T classification | T1/2 | 5(55.6%) | 4 | 1 |
|  | T3 | 26(54.2%) | 22 |  |
| N classification | N0 | 14(53.8%) | 12 | 1 |
|  | N1 | 17(54.8%) | 14 |  |
| Lymphatic vessel invasion | ly0 | 8(44.4%) | 10 | 0.39 |
|  | ly1/2 | 23(59.0%) | 16 |  |
| Venous invasion | v0 | 18(52.9%) | 16 | 1 |
|  | v1/2/3 | 13(56.5%) | 10 |  |
| Perineural invasion | ne0 | 3(60.0%) | 2 | 1 |
|  | ne1/2/3 | 28(53.8%) | 24 |  |
| Age | <65 | 9(56.3%) | 7 | 1 |
|  | ≧65 | 22(53.7%) | 19 |  |
| Sex | Male | 16(53.3%) | 14 | 1 |
|  | Female | 15(55.6%) | 12 |  |
| Tumor location | Head | 8(42.1%) | 11 | 0.26 |
|  | Body and/or Tail | 23(60.5%) | 15 |  |

Associations between IQGAP1 expression and the clinicopathologic characteristics were investigated. *P* values were calculated using the Chi-square test. T1, tumor limited to the pancreas, 2 cm or less in greatest dimension. T2, tumor limited to the pancreas, more than 2 cm in greatest dimension. T3, tumor extends beyond the pancreas but without involvement of the celiac axis or the superior mesenteric artery. N0, no regional lymph node metastasis. N1, regional lymph node metastasis. ly0, no lymphatic vessel invasion. ly1, mild lymphatic vessel invasion. ly2, moderate lymphatic vessel invasion. v0, no venous invasion. v1, mild venous invasion. v2, moderate venous invasion. v3, severe venous invasion. ne0, no perineural invasion. ne1, mild perineural invasion. ne2, moderate perineural invasion. ne3, severe perineural invasion.

**Supplementary File 1 Table 4.** Clinicopathological features of high and low ARL4C expression in PDAC with high IQGAP1 expression.

| **Parameters** |  | **High**  **ARL4C**  **cases** | **Low**  **ARL4C**  **cases** | ***P* value** |
| --- | --- | --- | --- | --- |
| Stage | IA-IIA | 11(78.6%) | 3 | 0.3 |
|  | IIB-III | 16(94.1%) | 1 |  |
| T classification | T1/2 | 3(60.0%) | 2 | 0.11 |
|  | T3 | 24(92.3%) | 2 |  |
| N classification | N0 | 11(78.6%) | 3 | 0.3 |
|  | N1 | 16(94.1%) | 1 |  |
| Lymphatic vessel invasion | ly0 | 7(87.5%) | 1 | 1 |
|  | ly1/2 | 20(87.0%) | 3 |  |
| Venous invasion | v0 | 15(83.3%) | 3 | 0.62 |
|  | v1/2/3 | 12(92.3%) | 1 |  |
| Perineural invasion | ne0 | 1(33.3%) | 2 | 0.037 |
|  | ne1/2/3 | 26(92.9%) | 2 |  |
| Age | <65 | 6(66.7%) | 3 | 0.063 |
|  | ≧65 | 21(95.5%) | 1 |  |
| Sex | Male | 15(93.8%) | 1 | 0.33 |
|  | Female | 12(80.0%) | 3 |  |
| Tumor location | Head | 20(87.0%) | 3 | 1 |
|  | Body and/or Tail | 7(87.5%) | 1 |  |

Associations between ARL4C expression in PDAC with high IQGAP1 expression and the clinicopathologic characteristics were investigated. *P* values were calculated using the Chi-square test. T1, tumor limited to the pancreas, 2 cm or less in greatest dimension. T2, tumor limited to the pancreas, more than 2 cm in greatest dimension. T3, tumor extends beyond the pancreas but without involvement of the celiac axis or the superior mesenteric artery. N0, no regional lymph node metastasis. N1, regional lymph node metastasis. ly0, no lymphatic vessel invasion. ly1, mild lymphatic vessel invasion. ly2, moderate lymphatic vessel invasion. v0, no venous invasion. v1, mild venous invasion. v2, moderate venous invasion. v3, severe venous invasion. ne0, no perineural invasion. ne1, mild perineural invasion. ne2, moderate perineural invasion. ne3, severe perineural invasion.

**Supplementary File 1 Table 5.** The sequences of ARL4C ASOs used in this study.

| ASO | Sequence |
| --- | --- |
| Control ASO | T(Y)^a^g^A(Y)^g^a^G(Y)^t^a^5(Y)^c^c^A(Y)^t^c |
| ARL4C ASO-1316 | G(Y)^5(Y)^A(Y)^t^a^c^c^t^c^a^g^g^T(Y)^A(Y)^a |

Lower case=DNA; N(Y)=AmNA; 5(Y)=AmNA_mC; ^=Phosphorothioated

**Supplementary File 1 Table 6.** List of antibodies used in this study.

|  |  |  | Application (Dilution ratio) | | |
| --- | --- | --- | --- | --- | --- |
| Antigen | Company | Catalog # | WB | IHC | ICC |
| ARL4C | Atlas Antibodies (Bromma, Sweden) | #HPA028927 | 1:1000 | 1:50 |  |
| Clathrin | BD Biosciences (San Jose, CA, USA) | #610500 | 1:1000 |  |  |
| EGR1 | Cell Signaling Technology (Beverly, MA, USA) | #4153S | 1:1000 |  |  |
| β-catenin | BD Biosciences (San Jose, CA, USA) | #610154 | 1:1000 |  |  |
| Ras (G12D) | Cell Signaling Technology (Beverly, MA, USA) | #14429S | 1:1000 |  |  |
| Hsp90 | BD Biosciences (San Jose, CA, USA) | #610419 | 1:1000 |  |  |
| HA | BioLegend (San Diego, CA, USA) | #901502 | 1:1000 |  |  |
| GFP | Life Technologies/Thermo Fisher Scientiﬁc (Carlsbad, CA, USA) | #A6455 | 1:4000 |  |  |
| GFP | Santa Cruz Santa Cruz Biotechnology  (Dallas, TX, USA) | #sc-9996 | 1:1000 |  |  |
| FLAG | WAKO (Tokyo, Japan) | #014–22383 | 1:1000 |  |  |
| IQGAP1 | Santa Cruz Santa Cruz Biotechnology  (Dallas, TX, USA) | #sc-376021 | 1:1000 | 1:800 | 1:100 |
| MMP14 | Abcam (Cambridge, UK) | #ab51074 | 1:1000 | 1:200 | 1:100 |
| Cytohesin2 | Proteintech Group, Inc (Chicago, IL, USA) | #67185-1-Ig |  |  | 1:100 |
| Paxillin | BD Biosciences (San Jose, CA, USA) | #610052 |  |  | 1:100 |
| FAK | BD Biosciences (San Jose, CA, USA) | #610087 |  |  | 1:100 |
| P-Paxillin  (Y118) | Cell Signaling Technology (Beverly, MA, USA) | #2541S |  |  | 1:100 |
| P-FAK  (Y397) | Life Technologies/Thermo Fisher Scientiﬁc (Carlsbad, CA, USA) | #44625G |  |  | 1:100 |
| Cortactin | Merck Millipore (Billerica, MA, USA) | #05-180 |  |  | 1:100 |
| Rac1 | BD Biosciences (San Jose, CA, USA) | #610651 | 1:1000 |  |  |
| Cdc42 | Cell Signaling Technology (Beverly, MA, USA) | #2466S | 1:1000 |  |  |
| CK19 | Abcam (Cambridge, UK) | #ab52625 |  | 1:100 |  |
| Mitochondria | Merck Millipore (Billerica, MA, USA) | #MAB1273 |  | 1:100 |  |
| LYVE-1 | Abcam (Cambridge, UK) | #ab14917 |  | 1:100 |  |
| YAP/TAZ | Cell Signaling Technology (Beverly, MA, USA) | #8418S |  |  | 1:100 |

WB, western blotting; IHC, immunohistochemistry; ICC, immunocytochemistry

**Supplementary File 1 Table 7.** Target sequences for siRNA used in this study.

| Gene | Sequence |
| --- | --- |
| Randomized Control | 5'-CAGTCGCGTTTGCGACTGG-3' |
| human *IQGAP1#1* | 5'-GCTGCACATAGTTGCCTTT-3' |
| human *IQGAP1#2* | 5'-CCCTAATGTAGAATGTCAT-3' |
| human *CYTH2#1* | 5'-GGATGGAGCTGGAGAACAT-3' |
| human *CYTH2#2* | 5'-GCAGTTTCTATGGAGCTTT-3' |
| human *ARPC2#1* | 5'-GCCTATATTCACACACGTA-3' |
| human *ARPC2#2* | 5'-CCTATATTCACACACGTAT-3' |
| human *MMP14#1* | 5'-GCAGCCTCTCACTACTCTT-3' |
| human *MMP14#2* | 5'-CCGACATCATGATCTTCTT-3' |
| human *KRAS#1* | 5'-GCATCATGTCCTATAGTTT-3' |
| human *KRAS#2* | 5'-GTTGGAGCTGATGGCGTAG-3' |
| human *CTNNB1#1* | 5'-CCCACTAATGTCCAGCGTT-3' |
| human *CTNNB1#2* | 5'-GCATAACCTTTCCCATCAT-3' |

**Supplementary File 1 Table 8.** Primer sequences for quantitative PCR used in this study.

| Gene | Forward | Reverse |
| --- | --- | --- |
| human *GAPDH* | 5'-TCCTGCACCACCAACTGCTT-3' | 5'-TGGCAGTGATGGCATGGAC-3' |
| human *B2M* | 5'-TGCTGTCTCCATGTTTGATGTATC-3 | 5'-TCTCTGCTCCCCACCTCTAAG-3' |
| human *ARL4C* | 5'-AGGGGCTGTGAAGCTGAGTA-3' | 5'-TTCCAGGCTGAAAAGCAGTT -3' |
| human *ARPC2* | 5'-AGATTTCGATGGGGTCCTCT-3' | 5'-CCGGAAGATTTTCAAGGTCA-3' |

*B2M*, *β2-microglobulin*.
